# Implicit–explicit gradient of nondual awareness or consciousness as such

**DOI:** 10.1093/nc/niab031

**Published:** 2021-10-08

**Authors:** Zoran Josipovic

**Affiliations:** Psychology Department, Graduate School of Arts & Sciences, New York University, New York, NY 10003, USA; Nonduality Institute, Woodstock, NY 12498, USA

**Keywords:** nondual awareness, consciousness as such, neural correlates of consciousness, meditation, consciousness

## Abstract

Consciousness is multi-dimensional but is most often portrayed with a two-dimensional (2D) map that has global levels or states on one axis and phenomenal contents on the other. On this map, awareness is conflated either with general alertness or with phenomenal content. This contributes to ongoing difficulties in the scientific understanding of consciousness. Previously, I have proposed that consciousness as such or nondual awareness—a basic non-conceptual, non-propositional awareness in itself free of subject-object fragmentation—is a unique kind that cannot be adequately specified by this 2D map of states and contents. Here, I propose an implicit–explicit gradient of nondual awareness to be added as the z-axis to the existing 2D map of consciousness. This gradient informs about the degree to which nondual awareness is manifest in any experience, independent of the specifics of global state or local content. Alternatively, within the multi-dimensional state space model of consciousness, nondual awareness can be specified by several vectors, each representing one of its properties. In the first part, I outline nondual awareness or consciousness as such in terms of its phenomenal description, its function and its neural correlates. In the second part, I explore the implicit–explicit gradient of nondual awareness and how including it as an additional axis clarifies certain features of everyday dualistic experiences and is especially relevant for understanding the unitary and nondual experiences accessed via different contemplative methods, mind-altering substances or spontaneously.

## Introduction

### Relevance of consciousness as such and its gradient

Consciousness as such has been a perennial concern of the nondual contemplative traditions for many centuries (Radhakrishnan and Moore 1967; Rabjam 2007). Recently, this topic has gained increased traction in both neuroscience and philosophy of mind. It has been suggested that a number of current impasses in understanding consciousness could benefit from including consciousness as such in research and theorizing (Koch *et al.* 2016; Josipovic 2019; Michel *et al.* 2019; Metzinger 2020; Baars 2021; Lepauvre and Melloni 2021).

The present discussion will first outline what consciousness as such is and then propose that the implicit–explicit gradient of consciousness as such can be added as the third dimension to the standard two-dimensional (2D) map of consciousness. This gradient informs about the degree to which consciousness as such is manifest in any experience, independent of the specifics of global state or local content. I will also explore how including this gradient clarifies certain features of everyday dualistic experiences and may be especially relevant for understanding the unitary and nondual experiences accessed via different contemplative methods, mind-altering substances or spontaneously.

Consciousness as such or nondual awareness will be discussed from its phenomenal, functional and neural levels. Other levels, such as ontological, metaphysical, ethical or soteriological, although no less important, will not be discussed here. The nondual view presented here sees consciousness and the brain as being like two sides of a coin, the same in being one and different in not being reducible to one another, with the indeterminate unknowing substrate akin to seeing the coin from its side in terms of probabilities only. Phenomenal descriptions of how nondual awareness appears in experience ground the present discourse. Functions of nondual awareness are discussed in terms of its role in experience and in terms of differentiating it from global states and local contents and from attention or monitoring with which it is frequently conflated. The neural level is discussed in terms of networks in the brain and their global organization.

### Terms and caveats to be read before proceeding

A caution about language is needed before proceeding. Language is linear, dualistic and temporal, with a subject-object structure, while consciousness as such is experienced as nondual, wholistic and atemporal, so discourse on this topic can easily appear circular and paradoxical, like attempting to describe a three-dimensional (3D) sphere using a one-dimensional line.

A frequent source of misunderstandings comes from the way this topic is presented. In order to point to what consciousness as such is, it first needs to be emphasized how it is different from states, contents and functions. Likewise, certain methods developed to enable one to recognize it, rely on separating and isolating consciousness as such from all other content. But then, it also needs to be pointed out that it can co-occur with any state and content. This can lead either to a mistaken impression that all content must cease for consciousness as such to be explicitly present or to conflating consciousness as such with changes in contents and states (Josipovic and Miskovic 2020; Metzinger 2020).

A number of terms commonly used in contemporary discourse on consciousness can have different meanings when applied to consciousness as such. Below, I list a few such terms and define how they will be used here.

#### Consciousness, awareness and consciousness as such

Consciousness refers here to conscious experience as a whole, which includes phenomenal, access, self-consciousness, etc. (Block 2007; Lau and Rosenthal 2011). Conscious experience can be said to have two aspects: the modified consciousness consisting of contents, functions, states and the indeterminate substrate, and the unmodified consciousness or consciousness as such (Rabjam 2007; Josipovic 2019). The terms modified and unmodified have been adopted from Samkhya philosophy but are here used only as pointers, without any ontological or metaphysical implications (Radhakrishnan and Moore 1967). The term awareness is not used here in the ordinary sense, as an awareness of a specific stimulus or awareness as general alertness (Laureys and Tononi 2011). Rather, it is used only for consciousness as such, unless indicated otherwise by the context. Table 1 summarizes this.

**Table 1.** Consciousness as experience

*Modified consciousness:*
Phenomenal contents (perceptual, affective, cognitive)
Functions (attention, memory, decision, language, imagination, meta-cognition, etc.)
Global states (waking, dreaming, deep sleep, minimally conscious, altered, etc.)
Indeterminate (un)conscious substrate (non-recognition of consciousness as such; implicit dualistic structuring of experience into conceptually reified subject and object)
*Unmodified consciousness:*
Consciousness as such (nondual awareness, empty cognizance in itself devoid of content)

#### Consciousness as such or nondual awareness

The terms consciousness as such and nondual awareness are used interchangeably and stand for the unmodified aspect of conscious experience in itself, a basic non-conceptual nondual awareness, irrespective of the level of arousal or amount of content that may co-occur with it. This is unlike some theories where the term consciousness as such refers only to awareness isolated from all other content (Metzinger 2020). Such awareness is experienced as that which is, and has always been, conscious in all experiences. In other words, as consciousness itself or consciousness as such. However, whether this is true in an ontological sense is not known and although highly significant will not be addressed here.

Note that on this view, consciousness as such is first and foremost an awareness and not merely an indeterminate unknowing substrate, as in some models (Srinivasan 2020). The substrate only covers or obscures consciousness as such (for further details, see the section ‘How is nondual awareness obscured from itself?’). To avoid conflating the two, I will refer to consciousness as such as nondual awareness wherever possible.

Unless stated otherwise, the present discussion pertains only to conscious experience. What makes a specific state or content conscious or unconscious will not be addressed here. The gradient of nondual awareness discussed here is for the most part orthogonal to conscious–unconscious dichotomy, although, as we will see later, its axis intersects with axes for global state and local content at their mutual zero point. The terms global state, alertness and arousal are used here interchangeably (for disambiguation, see Metzinger 2020).

#### Reflexivity, self-knowing, self-recognition and self-awareness

The term reflexivity as ordinarily used implies a conceptual meta-cognition and re-representation (Rosenthal 2012; Peters 2015). Here, however, it is used to refer to the inherent non-conceptual reflexivity of consciousness as such, awareness knowing itself to be aware non-transitively, as its property. This non-representational reflexivity has also been termed self-knowing and its activation a self-recognition (Kshemaraja and Singh 1990; Rabjam 2007). Such awareness is then termed the self-knowing awareness or self-awareness for short (Higgins 2011). This is different from how these terms are ordinarily used in psychology and neuroscience where they refer to knowledge, recognition or awareness of one’s self.

#### Representation and non-representation

Representation here refers only to conceptual mental representations or concepts for short, such as categorization, schema and various semantic, iconic and numeric symbols (Hubbard 2007; Shea, 2018). Conceptual categorization refers to categorization by labeling, as opposed to non-conceptual categorization by segregation or exclusion (Lamme 2020; Thompson 2021). The term non-representational or non-conceptual in this sense means not relying on such concepts, symbols or propositional beliefs. Debates between direct non-representational and indirect representational realism are outside of the scope of this discussion. Whether consciousness as such is entirely non-representational or only less representational remains an open question (Chalmers 2004; Zahidi 2014). On the view presented here, both non-representational and representational knowing are not only possible but necessary.

Importantly, the term representation does not here refer to neural representation, which will be termed neural correlate for disambiguation (Yuste 2015).

#### Duality and nonduality

Duality refers here to structuring of experience and knowing in terms of subject–object dichotomy. It also refers, more broadly, to the fragmentation of experience into conceptually reified polarities such as self–other, us–them, good–bad, internal–external and mind–body (Guenther 1984; Josipovic 2014, 2016). Nonduality then primarily refers to experiencing and knowing without such dualities. However, the term has also been used differently in other contexts, for example, to refer to an absence of phenomenal content in meditative absorption or to an absence of self-specifying process (Dor-Ziderman *et al.* 2016; Srinivasan 2020). In the present discourse, nonduality refers to how consciousness as such knows and abides with phenomenal contents and conscious states it co-occurs with. At an even deeper level, nonduality refers to the fully explicit nondual awareness appearing as simultaneously transcendent and immanent in contents and states that co-occur with it (Rabjam 2001; Josipovic and Miskovic 2020).

### Organization of the manuscript

For the sake of clarity and brevity, this discussion is organized in a top-down manner, emphasizing theoretical implications over empirical details of first-person accounts and neuroscience studies. However, ideas presented here are not merely normative. Phenomenal descriptions come from texts of various traditions, which form a body of evidence about centuries of experiences with various contemplative practices, and from increasingly large numbers of contemporary practitioners of meditation worldwide (Rabjam 2001; Maharshi 2006; Metzinger 2020). In addition, neuroscience studies of meditation, especially when paired with newly developing methods of assessing participants’ experience, have significantly increased our understanding of consciousness during contemplative and altered states (Tang *et al.* 2015; Petitmengin *et al.* 2019; Van Lente and Hogan 2020).

## Consciousness as such—nondual awareness

### What is it?

A currently popular view sees the brain as a computer-like device that lives in the dark about the body and the external environment, separated from them by the skull, and that the only way it can know them is by creating virtual, representation-based models and comparing their predictions with representations of inputs to minimize prediction errors (Seth 2015; Chalmers 2017). However, the brain is an integral part of the body, and so, it also knows the body and the external environment in a much more direct and intimate way, as its own lived reality, in which the internal and external environments are one unified, direct, non-representational experiencing or being (Varela *et al.* 1993; Thompson and Cosmelli 2011). It has been proposed that this more direct way of knowing may be instituted in living organisms via some type of resonance mechanism (Hunt and Schooler 2019; Levin 2021).

Consciousness as such is then a type of knowing, a basic non-conceptual, non-propositional awareness, without dualistic structuring of experience into conceptually reified subject and object. Hence, it has been termed nondual awareness (Rabjam 2001; Higgins 2011; Josipovic 2013, 2014; Dunne 2015; Laish 2015; Laukkonen and Slagter 2021).

Progressively deeper levels of meditation can reveal more subtle, ordinarily unconscious layers of conceptual mentation, so that at some point even the basic propositional beliefs and category concepts that specify subject and objects can cease, and yet a basic nondual awareness can still remain, vividly present and knowing (Lama 2004; MacKenzie 2015; Metzinger 2020). Granted, it is still possible that some entirely inaccessible unconscious conceptual processes, similar to those that construct ordinary cognitions, also underlie the phenomenal non-conceptuality of nondual awareness. In other words that only upper-level concepts and symbols cease but that deeper level conceptual and propositional processes remain the same. However, the absence of key properties of ordinary conceptual cognition and meta-cognition such as categorizing, memory associations, semantic tagging and especially the pervasive dualistic subject-object structuring makes it unlikely that nondual awareness is, in itself, a similar conceptual process as ordinary dualistic cognition (Higgins 2011; Thompson 2021). This is challenging because instances of completely isolated nondual awareness are relatively rare, and nondual awareness most often co-occurs with some amount of ordinary dualistic conceptual processes.

Although this awareness is ordinarily only implicit in experience, it can under certain circumstances become explicit. When explicitly present, it knows itself inherently to be aware and as the aware space within which conscious states and contents occur (Rabjam 2001; Josipovic 2019).

### Non-conceptual reflexivity

The key property of consciousness as such or nondual awareness is its inherent capacity to know that it knows, that is, to be aware and know that it is aware, without mediation by concepts, propositions, or semantic, iconic or numeric symbols (Rabjam 2007). This direct non-conceptual reflexivity is inherent in consciousness as such as its property and is non-transitive, without subject-object structure (Higgins 2011; Laish 2015; MacKenzie 2015; Fasching 2021). In nondual contemplative traditions, it has also been termed self-knowing, self-recognition or self-awareness and more recently as non-representational reflexivity (Ksemaraja and Singh 1990; Rabjam 2001; Josipovic 2019). This property makes nondual awareness phenomenally and functionally unique. In other words, consciousness as such is unique because it is precisely just that which knows and knows that it knows, directly, as what it is. It could then be said that, unless an organism or a system can, in principle, be reflexively aware in this way, it is not conscious in the same way that we humans are (for a more detailed discussion, see Josipovic 2019).

The inherent reflexivity of nondual awareness can become activated so that awareness recognizes itself and becomes explicit in experience with varying amounts of phenomenal content. For example, this self-recognition can occur when nondual awareness is isolated from other content during minimal or minimized phenomenal experience, such as instances of attaining lucidity during deep, non-REM (NREM) sleep. It can also occur with full phenomenal content during ordinary wakefulness or in altered states (Laish 2015; Dunne *et al.* 2019; Josipovic and Miskovic 2020; Metzinger 2020).

When nondual awareness becomes explicit during minimal phenomenal experience as in full meditative absorption, it appears as an open, empty cognizance, aware and present, but without any thoughts, emotions or perceptions, without a sense of body, orientation, time or the usual sense of self (Thompson 2014; Josipovic 2019; Metzinger 2020). Alternatively, various amounts and types of reduced content can be present during absorption states, with nondual awareness explicitly manifest (Metzinger 2020; Josipovic and Miskovic 2020).

When nondual awareness is fully explicit during wakefulness, it is experienced as simultaneously transcendent and immanent in conscious states and contents. It is transcendent, as the silent aware space that pervades and encompasses the entire conscious experience, one’s entire perceptual bubble; and also, immanent, as that out of which everything is made, the way water in a glass is both the medium in which ice cubes float and the substance out of which they are made (Radhakrishnan and Moore 1967; Rabjam 2001; Josipovic 2019).

### Dimensions or properties of nondual awareness

When nondual awareness is explicitly present, a number of its properties or dimensions can be self-evident. In the Asian nondual contemplative traditions, these have been usually stated as three or four but more can be differentiated depending on various criteria and interests (Rabjam 2007; Higgins 2011, 2019; Josipovic 2019; Metzinger 2020; Fasching 2021). They can be summarized in a list as:

- Being or presence—the obvious fact of awareness being present or phenomenally existing.
- Emptiness—an absence of conceptually assigned identity and conceptualizations about itself or phenomena that reify awareness as the subject and phenomena as objects essentially separate from it.
- Nonduality—a corollary of the above, without subject–object structuring of experience.
- Luminosity or radiance—a cognitive property appearing as the clear transparent light by which it knows itself and phenomena present to it.
- Non-conceptual reflexivity—a corollary of the above, refers to nondual awareness inherently knowing itself to be aware without relying on mediation by conceptual mental representations.
- Bliss—silent contentment of being entirely complete in itself, with no sense of any lack or any need for anything outside of itself, so in this sense, without intention.
- Singularity or unity—nondual awareness is singular and homogenous, a unity of all its dimensions, not compounded or constructed from them or from anything else.
- No self/self—without a constructed self, but the self-same awareness in all experiences, hence also termed the self.
- Boundless, timeless spaciousness—single aware space, in itself without edges or boundaries, the background context of any experience, pervading and encompassing both internal and external environments, without a psychological sense of time.
- Ecstatic pleasure—near orgasmic-like enjoyment of contact between nondual awareness and phenomena, beyond pleasure–pain dichotomy. Although specific pleasures and pains occur and are experienced as such, the underlying nature of experiencing is an ecstatic union of nondual awareness and any perceptual, affective or cognitive content.

### Functions of nondual awareness

In terms of its function, nondual awareness has been compared to a mirror, while phenomena that appear to it have been compared to reflections in the mirror (Norbu 2013). Its manner of knowing phenomena can be described as mere reflecting or registering, without categorization or further conceptual elaboration, i.e., without labeling, associating, evaluating, forming decisions or taking itself as a reified subject that knows phenomena as reified objects (for a detailed discussion and disambiguation from bare non-conceptuality, see Thompson 2021). Thus, nondual awareness is neither the reflective nor the pre-reflective consciousness as usually understood, neither slow nor fast thinking, as these are based on conceptual processes and on implicit subject–object duality (Zahavi 1998; Kahneman 2011; Thompson 2021).

Furthermore, just as a single mirror reflects all of the images of objects present in front of it, the effect of this singular awareness is to further unify various elements of conscious experience (Rabjam 2001; Blackstone 2007). This corresponds to a major function of nondual awareness in unifying intrinsic self-related and extrinsic environment-related aspects of experience (Josipovic *et al.* 2012). In this sense, nondual awareness, when explicitly present, functions as the conscious space in which experience occurs (Rabjam 2001; Josipovic 2019). Not the conceptualized schematic space of distances, routes or boundaries, but the single non-conceptual space encompassing the entire perceptual bubble of any nondual conscious experience, pervading the internal and external environments at the same time (Rabjam 2001; Blackstone 2007; Josipovic 2014). In such experiences, nondual awareness also functions to relax the habit of sorting experience into acceptable and unacceptable parts (Guenther 1984; Josipovic 2013).

### Neural correlates of nondual awareness

It is possible that nondual awareness is phenomenally and functionally unique but not neurally unique and that its neural correlates are the same as those for attention or arousal or their predictive models (Raffone and Srinivasan 2010; Webb and Graziano 2015; Metzinger 2020; for further details, see Josipovic 2019). Be that as it may, I will here present a view, as I have done in the past, according to which nondual awareness is also neurally unique (Josipovic *et al.* 2012; Josipovic 2014, 2019).

A neural correlate of nondual awareness needs to be able to function with both low and high levels of arousal and content and also serve as the integrative conscious space within which contents occur. A parieto-frontal cortico-cortical and thalamo-cortical network with a self-sustaining oscillatory resonant dynamic regime could perform such a function (Baars *et al.* 2013; Koch *et al.* 2016; LaBerge and Kasevich 2017; Heeger and Mackey 2019; Mashour *et al.* 2020). The central precuneus network fulfills these criteria for the neural correlate of nondual awareness (Josipovic 2014, 2019). This functional network links the central precuneus with the dorso-lateral prefrontal cortex (dlPFC, BA 9/10/46), the dorsal anterior cingulate and dorso-medial prefrontal cortex (dACC and dmPFC, BA32, BA8), and the inferior parietal lobe and temporo-parietal junction (TPJ), consisting of the angular gyrus (r/l-Ang,), supramarginal gyrus and the caudal superior temporal gyrus (Cavanna and Trimble 2006; Margulies *et al.* 2009; Cunningham *et al.* 2017; Buckner and DiNicola 2019).

The central precuneus is one of four or five functionally distinct areas of the precuneus, others being dorsal-anterior for somato-motor processing, dorsal-posterior for visuo-spatial, ventral posterior for autobiographic self and episodic memory, and the central for cognitive associative processes (Margulies *et al.* 2009). Further functional specialization may exist between left and right precuneus (Cavanna and Trimble 2006; Fingelkurts *et al.* 2020). The ventral precuneus, together with the posterior cingulate cortex (PCC) and retro-splenial cortex, is a part of the intrinsic system for processing self-related content, better known as the default mode network (DMN) (Fox *et al.* 2005; Buckner and DiNicola 2019). Recent studies have proposed that the ventral precuneus also has functional subdivisions, with the rostral ventral belonging to DMN proper and the caudal ventral belonging to the parietal episodic memory network (Zheng *et al.* 2020). The two dorsal precuneus areas, the anterior rostral and the posterior caudal, are not parts of the DMN; rather, they are related to the extrinsic system whose spontaneous resting-state activity is usually anti-correlated with that of DMN (Margulies *et al.* 2009).

Studies reporting activations and connectivity of the medial parietal lobe frequently conflate these four areas of the precuneus with each other or even the precuneus as a whole with the PCC (Brewer *et al.* 2011; Berkovich-Ohana *et al.* 2020). This blurring of functional differences also happens due to methodological reasons, such as when PCC is used to define DMN and spatial smoothing is applied in preprocessing (Margulies *et al.* 2009).

The central precuneus, located dorsally to the ventral precuneus, between the anterior rostral and posterior caudal dorsal areas, is unique among different subdivisions of the precuneus. It can adaptively associate with either DMN or the task-positive networks such as dorsal attention network (DAN) or the fronto-parietal network (FPN) for cognitive control (Li *et al.* 2019). This diversity functionally differentiates the central precuneus. While the ventral areas of precuneus link selectively to PCC and DMN, the anterior and posterior dorsal areas link selectively to left or right (FPN). Only the central precuneus can functionally connect with all of the networks, both the intrinsic and the extrinsic (Li *et al.* 2019). This corresponds to a major function of nondual awareness in increasing the integration of intrinsic self-related and extrinsic environment-related aspects of experience (Josipovic *et al.* 2012; Josipovic 2014). Note that, at this level, there is only one awareness, which can encompass either or both intrinsic and extrinsic contents. Since nondual awareness is the conscious space within which nondual experiencing occurs, its most likely neural correlate is the central precuneus and its network (Josipovic 2014).

When nondual awareness is explicit during normal wakefulness and its inherent self-knowing is vividly present, the prefrontal nodes of this network, dlPFC in particular, function to add the necessary amplitude and persistence to the network-wide resonance and coherence (Helfrich and Knight 2016; Schmidt *et al.* 2018). From the perspective of modular computational processing, this can be stated as the working memory maintaining the awareness-space online but without relying on representing or re-representing it via semantic, iconic, numeric or other concepts. At the same time, there will be a decrease of activity and connectivity between the central precuneus and angular gyrus of the right TPJ, reflecting the relaxation of conceptually reified body boundaries (Josipovic 2014; Blanke *et al.* 2015). When nondual awareness is isolated from other phenomenal content and abiding in its ground state, the activity of the central precuneus network may shift toward its posterior nodes (Ferrarelli *et al.* 2013; Josipovic 2019; Raccah *et al.* 2021).

According to the view presented here, the subcortical areas of brain stem and thalamus, including the reticular activating system and its cingulo-opercular projections, are necessary for the proper functioning of the central precuneus network, but they are not by themselves sufficient for nondual awareness. If they were, then all humans and even most mammals would be experiencing awareness that knows itself to be aware inherently. The fact, however, is that activating this direct self-knowing of awareness is challenging for most humans and in all likelihood impossible for animals.

The central precuneus network can be modeled as a macro-circuit or a neural net with extensive recurrent connections, which enable it to enter into sustained oscillatory resonance regimes following repeated ignitions (Buzsaki 2006; Moutard *et al.* 2015; Phillips *et al.* 2016; Heeger and Mackey 2019; Mashour *et al.* 2020). Such a network could be causally closed enough for awareness to perpetuate itself, given that the necessary level of arousal is maintained. The initial self-recognition of nondual awareness and its subsequent ongoing reflexive self-knowing correspond then to the network ignition and to subsequent stabilizing of the oscillatory resonance (Josipovic 2014, 2019). When nondual awareness is an ongoing presence, the central precuneus network will rest in its baseline, which is a tonic state of self-sustained oscillatory resonance stabilized in a sub-threshold low-power mode (Roach *et al.* 2018; Heeger and Mackey 2019).

In respect to nondual awareness, the main purpose of this organization is to enable the network to inform itself, and by extension the rest of the brain, about its state regarding its availability to process information, which, on this account, would be experienced as an empty cognizance or awareness that knows itself to be aware, and, at the same time, is open to knowing any information presented to it from the internal and external environments (Josipovic 2014, 2019). From this perspective, nondual awareness is a broadcast of the system’s current ground state, or in the language of the global workspace theory, the broadcast of its own broadcasting capacity (Baars *et al.* 2013; Ricard and Singer 2017).

When nondual awareness is explicitly present in the foreground of experience, the central precuneus network functions as the dynamic core of such global broadcast, which will also include local networks for contents that co-occur with it. When nondual awareness is implicit, the reduced activity of the whole network or the fragmented activity of its nodes are added to the broadcast as frames (Baars *et al.* 2013; Josipovic 2014, 2019; Godwin *et al.* 2015).

Of course, a neural network informing itself about its capacity to process information can be instituted in a relatively simple electronic circuit, without any sign of awareness or consciousness, so the biological constraints on a system’s capacity for consciousness apply here and so much more for nondual awareness that requires a human-level brain (Cook *et al.* 2014; Dehaene *et al.* 2017; Koch 2019).

### Differentiating nondual awareness from states and contents

The mirror metaphor can be also used to illustrate how nondual awareness is different from global states and local contents, provided we keep in mind that it is only a metaphor and not a mechanistic explanation. In respect to the global state, the level of arousal and alertness can be compared to the amount of illumination in the room, so that some minimum amount is necessary for the mirror to reflect anything, but aside from that, changing the level of illumination affects only how images appear in the mirror and not the mirror itself (Josipovic 2016). Examples of dissociation between awareness and global state are the instances of nondual awareness becoming explicit during lucidity in rapid eye-movement (REM) dreaming, or in deep, NREM sleep (Thompson 2014; Voss *et al.* 2014) and the near-death experiences (Martial *et al.* 2020). In those states, awareness can be present with low levels of arousal. Conversely, there are vegetative states of nonresponsive wakefulness without awareness (Boly *et al.* 2012). Awareness can also be present in hypomanic states of high arousal, such as those induced by psychedelics (Millière *et al.* 2018). In principle, nondual awareness can become explicit in all and any of these global states. Conversely, all those levels of arousal can and do occur without nondual awareness ever being realized. In other words, global state and its related arousal level do not specify nondual awareness.

Nondual awareness can also be differentiated from local contents. It can be present independent of the amount of content, with a large amount of content during full normal wakefulness or with a minimal content as in lucidity during NREM sleep (Josipovic and Miskovic 2020; Metzinger 2020). To regard isolated nondual awareness primarily as a minimal phenomenal experience, rather than as a unique kind, means that it is still seen as a content of sorts rather than that which is aware of content (Josipovic 2019). Treating awareness and content as differentiable has been accused of leading to the dead end of Cartesian dualism (Dainton 2002). However, this objection does not apply here because nondual awareness is inherently without subject–object structuring and abides non-dually with content (Rabjam 2001).

### How is nondual awareness obscured from itself?

At the most superficial level, what impedes self-recognition of nondual awareness is a simple ignorance, not knowing that there is such an aspect to human consciousness or that it can wake up to itself. Paradoxically, once the conceptual knowledge about nondual awareness is acquired, it can itself become an obstacle if one becomes too attached to it. It then interferes with one’s ability to be open to a more direct, non-conceptual experiencing and knowing (Rabjam 2007).

Nondual awareness is phenomenally the most subtle aspect of human consciousness, consciousness as such, and since normal conscious experiencing ordinarily operates at a much coarser level of conceptually solidified subject and objects, nondual awareness can be difficult to detect (Guenther 1984; Blackstone 2007). The attentional habit of focusing exclusively on perceptual, affective or cognitive contents, rather than also on that which is aware of such contents, also contributes to keeping nondual awareness implicit only.

Nondual awareness is also the most intimate aspect of experience—who one is as a conscious, aware presence in all one’s experiences—so that the ways in which one is defensively distancing from one’s authenticity contribute to it remaining hidden and implicit. The non-preferential, all-encompassing mode of knowing and experiencing that characterizes nondual awareness can trigger psychological defenses that keep unacceptable and threatening aspects of one’s experience from one’s conscious self, so that, at a subconscious level, allowing nondual awareness to become explicit may be experienced as threatening (Blackstone 2007; Lindahl *et al.* 2017).

Nondual contemplative traditions point to an even deeper level, at which nondual awareness is obscured from itself by the unconscious indeterminate substrate, or store-house consciousness, which is thought to function as a container for storing memories, akin to the psychodynamic notion of the unconscious (Germano and Waldron 2006; Higgins 2019). In itself, the substrate consists of three types of mistaken, usually unconscious, cognitions: first, a conceptual categorizing and reifying of nondual awareness as essentially separate from the rest of experience; second, the dimming of awareness’ cognitive luminosity so that nondual awareness fails to recognize itself; and third, mistaking such dimmed awareness for a conceptually reified subject and phenomena for reified objects (Rabjam 2007; Higgins 2019). Patterns of organizing experience along this dualistic subject–object polarity, from basic propositional beliefs and categorizations to elaborate self-world models, become stored and used as predictions, in other words, projected onto current experience (Wallace 2007; Josipovic 2016; Higgins 2019). The unconscious identification with the dualistic self-world model perpetuates this process (Metzinger 2010). Until nondual awareness self-recognizes, this substrate underlies and dualistically structures both conscious and unconscious states and contents, so that, e.g., attaining lucidity in REM or NREM sleep, does not automatically constitute the self-recognition of nondual awareness (for further discussion see, Josipovic and Miskovic 2020).

Nondual awareness and the substrate have been traditionally compared to the sun and clouds, respectively (Rangdrol 1990). A cloud covering can entirely obscure the sun, yet the sun is still present, and its light illuminates all on the ground below. While not visible, the sun can only be inferred on the basis of its light. As the clouds disperse, the sun is revealed, but it is not the case that clouds created or became the sun, much as the unconscious substrate only obscures consciousness as such, and does not create it or become it (Rangdrol 1990; Rabjam 2007; Higgins 2019).

The substrate has been referred to as the ignorance i.e. without beginning but that can have an end (Rabjam 2007). Normal development does not result in nondual awareness becoming spontaneously explicit. This holds true even in children who may have had some unitary or spiritual experiences early on. Rare exceptions are known to happen and then only in the late teens or early adulthood (Ray 1986; Maharshi 2000). In light of contemporary neuroscience, this can be understood as being due to the late myelination of long-range axonal connections between nodes of the central precuneus network (Miller *et al.* 2012; Huntenburg *et al.* 2018). However, once developed, this network still needs to be turned on or ignited for nondual awareness to be manifest explicitly (Josipovic 2019).

## The implicit–explicit gradient of nondual awareness or consciousness as such

**Figure 1.**
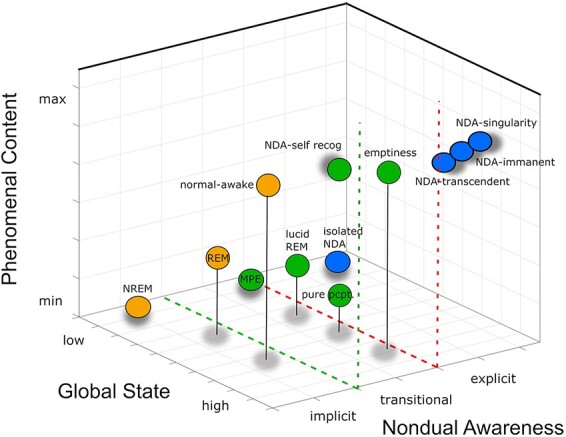
Implicit–explicit gradient of consciousness as such (nondual awareness) on z-axis. Two axes of the standard map, the global state on x-axis and phenomenal content of y-axis, with the gradient of consciousness as such or nondual awareness represented on z-axis. Three main zones of the gradient: implicit—orange, transitional—green, and explicit—blue. Specific experiences are represented as colored circles with gray shadows indicating their approximate locations on the gradient, for illustration purposes only. See below for details. Image courtesy of V. Miskovic

The gradient of nondual awareness informs about and maps how nondual awareness appears in any experience, independent from the global state and local content of that experience (see Fig. 1). In that sense, the gradient is related to temporary states, rather than to enduring traits. On the view that nondual awareness is self-same and unchanging, the gradient represents the degree to which the substrate obscures nondual awareness. In other words, the gradient represents how self-recognized the awareness is.

The most commonly used map of consciousness decomposes consciousness into two dimensions: the global state or level that indicates a degree of alertness on one axis, and the local content indicating a degree of vividness and richness or amount of perceptual, affective or cognitive content (Laureys and Tononi 2011). Since consciousness as such, as discussed above, is in principle dissociable from states and contents, it cannot be adequately specified with this standard 2D map. Instead, its implicit–explicit gradient needs to be added on the z-axis.

This gradient can be thought of as having three main zones or clusters, implicit, transitional and explicit, which are both discrete and continuous. Below I outline how a selection of experiences, both ordinary dualistic, and more unitary and nondual, can be understood in light of the implicit–explicit gradient and its map.

### Implicit zone of the gradient

In the first zone of the gradient, nondual awareness is present only implicitly. When nondual awareness is implicit, it is obscured from itself by the substrate’s mistaken cognitions and misidentifications as conceptually reified subject and object (Germano and Waldron 2006; Higgins 2011; Metzinger 2020). Its direct non-conceptual knowing becomes obscured by the indirect conceptual symbolic-based transitive thinking. The property of being-presence becomes obscured by the reified notion of existence and by grasping for it. The property of emptiness becomes obscured by the reified notion of nonexistence and by the fear of it. The property of bliss of being complete in itself becomes obscured by craving and reward-seeking (Guenther 1984; Rabjam 2007).

This organization of experience has been termed duality (Josipovic 2014; Dunne 2015). It fragments experience into opposing poles, such as mind vs. body, inside vs. outside, self vs. other, us vs. them, etc., which in traditional analysis cause pervasive unnecessary discontent and suffering (Dahl *et al.* 2015; Josipovic 2016). On this view, the more implicit nondual awareness is, the more dualistic and fragmented the experience is, or in the language of prediction theories, the more it becomes covered up by the self-world model and its predictions (Metzinger 2020; Laukkonen and Slagter 2021). A range of duality can be found both within healthy experience and within clinical conditions. When an experience is one of comfort and ease, there will be less dualistic fragmentation and the properties of nondual awareness, although still known only indirectly, will be more apparently reflected in experience. For example, being-presence will be reflected as peacefulness, awareness as an abatement of fixated beliefs, bliss as enjoyment and happiness, and singularity as authenticity and connectedness. When an experience is one of ongoing stress and struggle or when the survival mechanisms and ego-defenses are chronically overactive, the dualistic fragmentation will be more pronounced, and the nondual awareness and its qualities will usually be more obscured (Guenther 1984; Kohut 2009; LeDoux and Lau 2020). When nondual awareness is implicit only, locating an experience along its gradient cannot be determined directly. However, since the gradient represents a summation of different properties of nondual awareness, this can be estimated indirectly by assessing how much each property is reflected in an experience.

The more dualistic and fragmented an experience is, the less optimally integrated the global brain organization will be. This is most evident in clinical cases that show signatures of global disorganization, such as higher entropy and decreased functional segregation between resting-state networks, accompanied by the loss of their internal coherence (Sheffield and Barch 2016). On the other end of the disorder spectrum, global disorganization can be due to a hyper-synchronization, as in epileptic disorders (Kobayashi *et al.* 2017). Healthy conscious dualistic experiences, when nondual awareness is implicit, show the opposite signatures, a reduced entropy and more fixed attractor organization (Carhart-Harris and Friston 2019). For example, more distinctly defined attractors such as front vs. back, then, reflect the organization of experience into a conceptually constructed knower instituted in the PFC, which knows representations of objects in the posterior part of the brain. Similarly, a more rigid functional segregation of the resting-state networks can be found (Fox *et al.* 2005; Josipovic *et al.* 2012).

Given that the implicit zone of the gradient is characterized by nondual awareness not knowing itself directly, we can expect that this will be accompanied by a decrease in sustained oscillatory resonance and intra-network functional and effective connectivity in the central precuneus network, most significantly between the central precuneus and dlPFC.

### Transitional zone of the gradient

The second zone of the gradient is the transitional zone. It has two sections. The first one, related to a variety of unitary experiences that reflect more of the properties of nondual awareness, but without nondual awareness directly recognizing itself. The second one, in which nondual awareness recognizes itself directly as that which is aware.

#### Transitional unitary experiences

On this view, unitary experiences such as those encountered although contemplative practice, psychedelics or spontaneously, are possible because nondual awareness is present in the background of all experiences, even when unrecognized (Josipovic 2010, 2013). When the endogenous or exogenous conditions cause the dualistic subject–object self-world model to temporarily relax or cease, commonly reported as ego dissolution (Yaden *et al.* 2017; Barrett and Griffiths 2018), properties of nondual awareness can become more clearly reflected in experiences, giving them, among other properties, their unitary character (Rabjam 2001).

Interestingly, the more subtle the content, the more clearly it can reflect the properties of nondual awareness. For example, a more subtle experience of internal and external energy can reflect the unity property more strongly than the coarser experience of solidity of the body and objects in the environment. Further along in subtlety, an actual—not merely imaginary—formless experience, such as that of infinite light, can reflect the properties of nondual awareness to a great degree and with relatively little distortion.

A variety of experiences in this range will have the neural signatures of their chief characteristics. For example, depending on the degree, ego dissolution will show decreased activation and connectivity of areas involved in self-specifying processes such as DMN or the subcortical areas of the core self (Dor-Ziderman *et al.* 2016; Metzinger 2020).

However, while subjects may report awareness as part of their unitary experiences, the common characteristic of all experiences in this group is that they do not yet include the direct self-recognition of nondual awareness by itself (Hanley *et al.* 2018, Josipovic 2019).

#### Transitional self-recognition

The second section of the transitional zone of the gradient is related to the initial awakening of nondual awareness to itself. This is the key aspect of the gradient, the activating of the self-knowing property of consciousness as such, its non-conceptual reflexivity.

A number of different factors can contribute to an optimal situation for the self-recognition to activate. Both traditional and contemporary accounts indicate that these can vary considerably between individuals and situational contexts (Rangdrol 1990; Maharshi 2000; Metzinger 2020). What actually causes the ignition of this auto-reflexivity is not known, other than it is a spontaneously occurring event and that some cognitive strategies, like attending to awareness, as in awareness-of-awareness practices, or questioning, as in self-inquiry practices, can facilitate it (Josipovic and Miskovic 2020; Maharshi 2006).

Two parallel cognitive processes co-occur as the direct non-conceptual reflexivity activates and self-recognition ignites: first, the conceptual processes that construct the multi-layered reified subject and objects temporarily relax or cease, so that awareness emerges and is present however briefly, relatively unobscured, but long enough to unglue itself from identification with the constructed self; and second, nondual awareness wakes up to itself and ‘sees its own face’, as traditionally expressed (Rabjam 2001). It becomes aware that it is aware directly and not via conceptualizations about itself (Ksemaraja and Singh 1990; Rabjam 2007; Laish 2015; Josipovic 2019).

Interestingly, the relation between these two cognitive processes is asymmetric. While the ignition of this intuitive leap is the sine-qua-non of self-recognition, the abatement of self-specifying mental representations and even of dualistic subject–object structuring is not. Paradoxically, nondual awareness’ knowing of itself can ignite even with the presence of dualistic subject–object conceptualizations, as Saraha famously stated:

the radiance of self-awareness in awareness and un-awareness…shines without removing the grime of subject and object.

(Saraha, as quoted in Higgins 2006)

The neural correlates of the activation of the inherent reflexivity of awareness will be related to the ignition in the central-precuneus network and to establishing a more sustained oscillatory resonance and coherence, especially between the central precuneus and lateral PFC nodes of this network, as previously proposed (Josipovic 2014, 2019). In such instances, we can also expect to see a decrease in activation and connectivity of the areas of the medial temporal lobe that are involved in conceptual representations and schema-based predictions, together with an accompanying decrease in the involvement of the semantic network areas (Josipovic 2019; Metzinger 2020; Laukkonen and Slagter 2021; Thompson 2021).

Nondual awareness can appear to be intrinsic only as pure subjectivity or extrinsic only as empty of any self. However, in itself, nondual awareness is neither exclusively intrinsic nor extrinsic. Since it can pervade and encompass both types of content, either alone or together, it is transcendent to them, similarly to how space is transcendent to objects in it. In principle, neither an intrinsic content like introspection nor an extrinsic content like an absorbing perceptual experience are in and of themselves antithetical to nondual awareness self-recognizing and becoming explicit.

### Explicit zone of the gradient

The explicit zone of the gradient is characterized by the self-knowing nondual awareness being fully in the foreground of experience. It has three sections.

#### Nondual awareness as transcendent

The first section is one of realizing the identity of nondual awareness and space, in which nondual awareness becomes established as the ongoing space-like context, and all states and contents irrespective of how they are constructed, are experienced within it, as within a metaphoric ground of being (Rabjam 2001; Blackstone 2007; Higgins 2011).

#### Nondual awareness as immanent

The second section of explicit zone of the gradient is one in which nondual awareness appears as immanent within contents and states, as that which they are made of, and all its properties are reflected clearly in them (Radhakrishnan and Moore 1967; Rangdrol 1990).

#### Nondual awareness as singularity

The third section of the explicit zone is that of unity, when nondual awareness appears as both the aware space in which conscious states and contents occur and as the substance out of which they are made, so it is experienced as simultaneously transcendent and immanent in experience (Josipovic 2016, 2019). At the full unity, both nondual awareness as the ground and all contents and states within it are a singular, spontaneously occurring authentic presence (Klein and Wangyal 2006).

Neural correlates of explicit nondual awareness will be seen in the effects of the central precuneus network on the global organization of the brain, in the increased functional integration of intrinsic and extrinsic networks and in the increase in left–right symmetry (Lutz *et al.* 2004; Josipovic *et al.* 2012; Johnson 2016). Akin to psychedelic states, explicit nondual awareness will exhibit global dynamic signatures of increased criticality, poised between too much disorganization and too much organization, and accompanied by the relaxation of more fixed attractor states, such as front vs. back ones (Millière *et al.* 2018; Carhart-Harris and Friston 2019).

Perception co-occurring with nondual awareness during normal wakefulness shows less top-down prediction, corresponding to subjects reporting less memory associations and conceptual interpretations tagging onto perceptions (Fucci *et al.* 2018; Laukkonen and Slagter 2021). Relaxation of different layers of conceptually constructed self, a common effect of many contemplative practices, occurs with nondual awareness as well, although attenuation of DMN may be less pronounced than in focused attention or open monitoring styles meditations, as nondual awareness can non-preferentially contextualize any type of content (Dahl *et al.* 2015; Dor-Ziderman *et al.* 2016; Josipovic 2016). On the level of body-based self, the relaxation of the reified representations of body boundary may be seen in decreased connectivity between the central precuneus and the angular gyrus in the right temporo-parietal area (Josipovic 2014; Blanke *et al.* 2015).

## Discussion

### Implicit–explicit gradient as z-axis

The most commonly used map decomposes consciousness into two dimensions: the global state or level that indicates a degree of alertness on one axis, and the local content indicating a degree of vividness and richness or amount of perceptual, affective or cognitive content (Laureys and Tononi 2011; Aru *et al.* 2019). Mapping consciousness in this way has been useful, especially in the field of neurology, but, as many have noted, rather incomplete (Overgaard and Overgaard 2010; Bayne *et al.* 2016). For example, features central to altered states of consciousness cannot be specified by a single dimension of level and require other dimensions (Bayne and Carter 2018; Millière *et al.* 2018). Contents too are better portrayed as a summation of a number of other dimensions or factors, such as subjective sense of specificity, vividness and intensity (Fazekas *et al.* 2020).

The standard 2D map of consciousness lacks the third dimension or z-axis. Various features of consciousness have been proposed as a possible z-axis, such as self and self-awareness (Hanoǧlu *et al.* 2014), subjective report of level or state (Bachmann 2012), and the connection with the external environment (Carhart-Harris *et al.* 2018). From the perspective presented here, the main issue with the 2D map is that it conflates consciousness as such or basic nondual awareness with global state and local content (Josipovic and Miskovic 2020).

Perhaps, the most accurate way of modeling nondual awareness would be as a multi-dimensional state-space, where its properties would be dimensions of the model. Each property could then be represented by a vector specifying its vividness, clarity, stability, etc. (Millière *et al.* 2018; Fazekas *et al.* 2020; Metzinger 2020). Such multi-dimensional state-space models better capture many of the nuances and complexities of consciousness (Berkovich-Ohana and Glicksohn 2014; Fazekas and Overgaard 2016).

However, the number of dimensions in such a model could increase rapidly, depending on one’s criteria for selecting them and how imaginative one may be in labeling them. This could easily lead to a considerable overlap between model’s vectors. Additionally, selecting and labeling a feature does not necessarily make it a genuine dimension of nondual awareness. Instead, it could be referencing a change in perceptual, affective and cognitive content or capacities, or in the level of arousal and alertness, that are occurring due to the presence of nondual awareness.

It has been proposed that each of the axes of the standard 2D map can be seen as a summation of various related dimensions of a multi-dimensional state-space model (Bayne *et al.* 2016). The z-axis, proposed here for a 3D map of consciousness, could similarly be seen as the summation vector of all of the dimensions of nondual awareness, represented as the implicit–explicit gradient that plots the degree to which nondual awareness is manifest in an experience.

A note of caution: Temptation needs to be resisted to take locations on the gradient too literally, as objectively existing levels, which one then thinks of as goals to attain, rather than as pointers indicating features of experience (Rabjam 2007). Both traditional and contemporary accounts of methods for realizing nondual awareness abound with descriptions of stages of progress, which can be understood in terms of trait acquisition, but are for the most part such pointers. When they are conceptually reified and affectively invested in, they can trap one on an endless treadmill of future goals to be attained in pursuit of some ideal of perfection—a special kind of hell.

### Differentiating consciousness as such, from functions of consciousness

Nondual awareness can be confounded with functions of consciousness that co-occur with it. Differentiating it from some functions, like language or mental imagination, is relatively straightforward, since nondual awareness is non-conceptual and non-symbolic. Differentiating it from other functions, like voluntary and involuntary attention, working memory and meta-cognition, can be more challenging (Dunne 2015; Josipovic 2019). This is compounded by the fact that these functions are frequently employed in various contemplative methods used to arrive at nondual awareness (Josipovic 2010, 2014; Dunne 2015; Josipovic and Miskovic 2020).

A key purpose of attention, whether voluntary or involuntary, is selection of content. In contrast, nondual awareness is choice-less or non-preferential in relation to content, akin to a mirror’s relationship with images reflected in it (Higgins 2011; Norbu 2013). Rather than preferentially selecting some content or its features as a foreground, nondual awareness encompasses an entire conscious experience equally. While being functionally different, attention and awareness overlap, so it can be difficult to differentiate them experimentally, as evidenced by efforts to disentangle consciousness as perceptual awareness from attention (Lamme 2004; Maier and Tsuchiya 2021).

Meditation methods that rely on attention cultivate focused attention and bare non-distractedness, both with and without an object of focus (Tsongkhapa 1996; Dunne 2015). These practices can often lead to absorption states with reduced levels of content. The shutting down of perceptual, affective and cognitive content is often confused with nondual awareness itself, but the resultant states of mental quiescence, non-distracted attention or even bare non-conceptuality are not in and of themselves nondual awareness or consciousness as such (Manjusrimitra and Lipman 2001; Josipovic and Miskovic 2020; Metzinger 2020). Neural correlates of such absorptions have been found in the global reduction of cortical activity and the slowing down of EEG signal frequencies (Berkovich-Ohana *et al.* 2015; Berman and Stevens 2015). Additionally, absorption in bare non-distractedness may be reported as an experience of isolated nondual awareness without content (Winter *et al.* 2020). The observed neural signatures of such a state are mainly in the areas of the DAN, indicating that rather than being a neural signature of pure nondual awareness itself, they most likely reflect the reliance on voluntary focused attention together with breath retention, to control and reduce the contents of one’s consciousness (Winter *et al.* 2020).

Monitoring and vigilance, which are, in addition to attention, also related to alertness and meta-cognition, are the primary functions employed in mindfulness meditation. Once a level of skill is reached that does not require effortful top-down control, the neural correlates of such monitoring have been found in the areas of the salience network and the related cingulo-opercular system for control of arousal and alertness (Lutz *et al.* 2008; Tang *et al.* 2015; Metzinger 2020). These areas have been proposed as the neural signature of pure awareness encountered in minimal phenomenal experience or absorption (Metzinger 2020). Most likely, they reflect the mechanisms for regulation of alertness rather than being the neural correlate of awareness itself (Sadaghiani and D’Esposito 2015). Finding of the anterior insula involvement in these instances can also be related to the method that involves tracking interoceptive signals, such as sensations of breath in the body (Farb *et al.* 2007). The global state can be changed by directly or indirectly manipulating the level of arousal in the brain, e.g., via stimulation of the basal forebrain, but given some minimal necessary arousal, nondual awareness is orthogonal to the level of arousal or alertness in the brain (Pal *et al.* 2020). Furthermore, in contrast to the integrative function of the central precuneus network, the salience network that includes the cingulo-opercular areas for the control of alertness is mainly involved in involuntary switching between the extrinsic and the intrinsic systems, thus contributing under normal conditions to their functional segregation (Menon and Uddin 2010). Interestingly, the central precuneus network has a shared node with the cingulo-opercular network in dACC (Margulies *et al.* 2009). Beyond this, differentiating nondual awareness and minimal phenomenal experience has been discussed extensively elsewhere and will not be repeated here (Josipovic 2019; Josipovic and Miskovic 2020).

Nondual awareness is also different from meta-awareness, a conceptual meta-cognition that one is aware. From a representational perspective, being aware that one is aware requires a re-representation of the higher-order representation of a first-order representation of being in a state of awareness (Rosenthal 2012). The necessity of such third-order re-representation seems like an obvious evidence that such conceptual processes are not the mechanism of nondual awareness, since the reflexivity of nondual awareness is, so to speak immediate, as its inherent property, and is non-conceptual and non-transitive, so phenomenally it is very different from attending as a subject to awareness as an object of one’s conceptual knowing (Rabjam 2007; Peters 2015; Josipovic 2019).

Some contemplative traditions train a non-propositional meta-awareness (Raffone and Srinivasan 2010; Dunne *et al.* 2019). This is still not consciousness as such, as the inherent self-knowing of nondual awareness is here intentionally inhibited in order to attend to qualities of one’s attention or to one’s perceptual and affective states (Dunne *et al.* 2019).

Unlike functioning of the working memory during conceptually based experience, during explicitly manifest nondual awareness, there is no intentional maintenance and manipulation of conceptual-symbolic representations of nondual awareness itself. However, to the extent that hippocampal–cortical associations are formed through any repeated experience, it is likely that some conceptual memory schemas related to nondual awareness and to events surrounding it will be formed and become triggered during a future occasion of nondual awareness. While nondual awareness itself is not a re-construct from memory, its presence does not interfere with memory functions. Thus, in practice, repeated experiences of nondual awareness are often a mix of actual nondual awareness and some constructed contents (Josipovic 2019; Metzinger 2020; Laukkonen and Slagter 2021). However, the global broadcast of nondual awareness does not require semantic tagging in working memory; in other words, the broadcast can include non-conceptual awareness and less conceptually constructed or even entirely non-conceptual content (Dehaene *et al.* 2017; Ricard and Singer 2017; Josipovic 2019).

According to predictive coding theories, much of what constitutes conscious experience are the outputs of various unconscious predictive models, such as those for interoception, attention or alertness (Seth 2015; Webb and Graziano 2015; Carhart-Harris and Friston 2019; Metzinger 2020). It then stands to reason that consciousness as such, as a basic non-conceptual nondual awareness, would have its own predictive model too. In other words, it may not be necessary to insist on specifying nondual awareness in terms of a predictive model of something other than itself. Furthermore, asking if a predictive model of awareness is alone sufficient for creating the experience of awareness is like asking if a higher-order thought alone is sufficient for a conscious experience of an object whose first-order representation it is re-representing (Brown *et al.* 2019). Even if it were, it would be merely virtual. In contrast, a major benefit of non-conceptual nondual awareness is in that it can free us from the matrix of a conceptually constructed virtual self and world.

## Limitation and future directions

The present discussion is grounded in a phenomenal level of description, an approach that has its inherent advantages and disadvantages. Due to limitations of space, some topics were not explored. For example, the way each dimension, or property, of nondual awareness appears in experience can be explored further in terms of how it changes along the gradient. Neural correlates of those changes can be specified. In light of this, the gradient can be explored further by situating a wider variety of experiences along its axis. This may be especially relevant for spiritual and contemplative experiences.

Future studies could test the expected global dynamic signatures of explicit nondual awareness by comparing them when it is isolated from contents vs. when it is explicit with a normal level of content in wakefulness, or with a high level of content as in some psychedelic states. It would be also interesting to see how a measure of the implicit–explicit gradient correlates with the phi measure of integration (Koch *et al.* 2016).

Finally, even though this paper presents a key aspect of human consciousness, it does not address the hard problem of consciousness or how the ideas presented here relate to current theories of consciousness. These will be topics for future explorations.

## Conclusion

Consciousness as such or nondual awareness is unique, and in trying to understand and research it, it should not be reduced to other aspects of consciousness, such as states and content or different functions or capacities. Including it into a research program on consciousness would help with current impasses in our understanding and lead to refinement of current theories or to new more encompassing theories of consciousness.

Mapping consciousness as such or nondual awareness onto the z-axis, as the gradient of how implicit or explicit it appears in experience, can add clarity to attempts to systematize unitary or mystical experiences. Various characteristics, which have been thought to give them their uniqueness, such as ego-dissolution or introvert–extrovert features, can now be further specified and related to how implicit or explicit nondual awareness is during an experience.

## Data Availability

There is no experimental data for this study, because the study was theoretical only.
